# Medical Education and the Public Health

**Published:** 1922-03

**Authors:** 


### MEDICAL EDUCATION AND THE PUBLIC HEALTH.

**(From a Correspondent.)**

MEDICAL education is a difficult and complex problem which demands constant attention on the part of the authorities of the General Medical Council. Reforms are not always easy to frame, and frequently are even more difficult to bring into practice, but there are subjects needing urgent alteration, which are under consideration at the present time, and it is to be hoped that improvements will not be unduly delayed.

The present medical curriculum consists of a preliminary year's work at Biology and Chemistry, followed by eighteen months at Anatomy and Physiology, and finally two or three years' clinical work. Recently, there has been an alteration whereby the work of the first year, or part of it, can be completed at school, before the university course is commenced ; the object of this is to enable more time to be spent at the later important stages, especially in the hospital at clinical work. This is an improvement, although it has really lengthened the course, as study for medical degrees commences at school and adds further to the time taken in obtaining a registrable qualification. There is an objection to this, in that it interferes with the general education at school, and it seems that much of this preliminary work could be deleted without danger to the efficiency of the graduate.

The second stage of Anatomy and Physiology is extremely important, as these two subjects respectively lay the foundation of surgery and medicine. To the surgeon a comprehensive knowledge of anatomy is essential, and only by a true understanding of the normal physiological conditions of the body can the clinician expect to become expert later on in the diagnosis and treatment of diseased states. This part of the curriculum cannot, then, be altered without untoward results.

Necessary as is the preceding phase of the university course, the last stage of undergraduate work—the preparation for the final examination—is the more vital, for it is during this time that the student comes into contact with clinical medicine and the allied subjects. Destructive criticism is very easy, and not always desirable, but there are several matters which cannot be overlooked, and they stand in urgent need of revision. The President of the British Medical Association, at the annual meeting in July last, stated that in his long experience of university education two factors stood out prominently ; he noticed, firstly, the extraordinary range of knowledge expected of the student presenting himself for his final examination, and, secondly, the inadequacy of the student's attainments, having regard to the serious and responsible duties awaiting him. There are many factors involved in bringing about this result, and perhaps one of the most important is the lack of proportion which is displayed in the teaching of the various subjects. Two or three years is not a long time for the student to obtain widespread knowledge, and the diversity of subjects tends to produce only a superficial knowledge in each ; this is not wanted, for in such subjects as midwifery and venereal diseases sound practical learning is essential, whereas less time could be given to other branches of work. For example, it is no exaggeration to say that frequently an afternoon at a clinical demonstration in the wards or an out-patient department is spent on the discussion of some curiosity as von Recklinghausen's disease, or an obscure nervous disorder. It matters little to the general practitioner whether he diagnoses such a disease or whether he fails to notice a commencing lenticular degeneration, or some pituitary abnormality of function, only determinable by abstruse laboratory tests ; but it is little short of disastrous, both to himself and to the public health, if the doctor is unable to diagnose and treat the ordinary emergencies of labour, or venereal disease and its complications, for such are the conditions which he will encounter every day in his practice.

Again, it is hardly necessary for the young graduate to be familiar with the exact details of the seven or eight operations for removal of the foot (and the names of the men who first described them), or even that he should be an expert refractionist or bacteriologist, and it seems that much of the time wasted in acquiring the intricacies of such subjects as these could be more profitably used in becoming familiar to a greater degree with the common pathological conditions found in the general practice of medicine.

The study of midwifery has been the subject of much comment, and there is urgent need for improvement in this section of the curriculum ; the time devoted to it is usually short, and the practical work is frequently insufficient to ensure a high standard of efficiency. It is difficult to suggest anything desirable other than an increase in the time spent on midwifery, and to augment the number of resident obstetric appointments. This latter could be accomplished if maternity homes are established all over the country for use by the poorer classes, and the resident medical officer could be guided in his work by a local consultant.

Another point to which attention is now being given is the subject of examinations. In these the anonymity of the student is preserved as far as possible, no notice is taken of his capacity or industry during his period of training, and the extent of his knowledge has to be determined by selected questions, written and oral (with a certain amount of practical work), all of which cannot cover the whole range of his studies. It seems, therefore, that a conscientious and unbiased report on the work of the student by his teachers should form an invaluable adjunct to the test of the examination in enabling the examiners to decide the result..

A most urgent subject is that of the teaching of Preventive Medicine in the curriculum. This very important branch of medicine is inadequately dealt with, and the average graduate has not the slightest idea of the value of it. Anything in connection with State Medicine is regarded by the student as something to do with drain-testing, sewage disposal water or supply, and it is not surprising that Public Health officials are suspiciously looked upon, as one book aptly states, as being " nothing more than dignified and overpaid plumbers." So much time is devoted to elaborate diagnosis and treatment that the preventive side is rarely heard of, and the student forgets the platitude that prevention is better than cure. There is no desire to criticise unjustly, but in so many diseases no drug is of any avail, and treatment is merely palliative, that if there is the slightest chance of prevention or beneficial early treatment these subjects should be considered at great length in the curriculum.

Having dealt with undergraduate education it is now appropriate to discuss shortly the important question of post-graduate education. The object of the student's career before qualification is to teach him how to learn, and the new graduate is in a position to make good use of this preliminary training by holding various hospital appointments. It is desirable that these should have some bearing on the future of the graduate, and those departments appertaining to the work intended to be followed should be chosen. As the recent Commission on Post-graduate Education pointed out that there are not enough appointments to go round, it is necessary that efforts should be made to increase them. Various courses of instruction on special subjects are now arranged at frequent intervals, and are of great value to those requiring further knowledge in different branches of medicine.

In addition to this, provision must be made for the general practitioner desirous of keeping in touch with new treatment and up-to-date methods. The report of the Commission, referred to above, recommends the formation of a well-equipped and Central Post-graduate Hospital and Medical School in London ; it is proposed to appoint consultants and senior resident officers, who would be responsible for teaching, and each of these men would receive an honorarium. The funds for maintaining such an institution would be obtained partly from fees, and partly from a grant from the State. It is difficult to see how the practitioner is to give up part of his annual holiday to obtain further knowledge, and the members of the Commission quite realise that the doctor's period of rest is of more importance than the extra skill he might acquire at a post-graduate course. A panel of substitutes has been suggested, but this does not seem practicable. In future, if there is to be, as seems probable, a surplus of doctors, it may be possible to arrange for partnership to consist of more members than at present, and then each partner in turn might obtain sufficient leave for both a holiday and some work at a post-graduate hospital.

Further, the recommendations include a general Institute of State Medicine, which would abolish the uneconomic system at present in vogue, whereby candidates for Public Health degrees study in at least four or five different schools of London University and at various other provincial universities. In addition to effecting this, the institute would arrange instruction in forensic medicine, in toxicology, in industrial medicine, as well as in the relation of the practitioner to the State, to his fellow-practitioners, and to the community at large. It seems, however, more than probable that the formation of both the Post-graduate Institution and the State Medicine College will have to be held in abeyance for a time.

### THE ROYAL SANITARY INSTITUTE.

### Bournemouth Congress.

Major-General the Rt. Hon. J. E. B. Seely, C.B., C.M.G., P.C., D.S.O., M.P., has consented to accept the office of President of the Thirty-third Congress of the Royal Sanitary Institute, to be held at Bournemouth from July 24 to 29.

Sectional Meetings will be held for the reading and discussion of papers relating to Sanitary Science (president, Sir Arthur Newsholme, K.C.B., M.D., F.R.C.P.) ; Engineering and Architecture (president, Sir Henry Tanner, C.B., I.S.O., F.R.I.B.A.) ; Maternity and Child Welfare, including School Hygiene (president, Sir George Newman, K.C.B., D.C.L., M.D., F.R.C.P.) ; Personal and Domestic Hygiene (president, Mrs. Muriel Lefroy) ; Industrial Hygiene (president, Mr. H. Gordon Selfridge).

A Health Exhibition illustrating municipal sanitation and domestic health and comfort will be arranged in connection with the meeting. Visits are arranged during the Congress to places of sanitary interest.

Fellows, Members and Associates of the Institute are supplied with tickets on application to the Secretary before the Congress. Tickets may also be obtained at the reception room during the meeting. To those not connected with the Institute, the price of tickets, entitling the holder to the use of the reception room, to admission to the Presidential and other addresses, to all meetings, to the Exhibition of the Institute, and copies of the Journal of the Institute containing the proceedings of the Congress, is £1 11s. 6d. each ; ladies' tickets (not including copies of the proceedings), 10s. 6d.

The Council invite papers on subjects relating to Health and Sanitary Science. Accepted papers are, as far as possible, printed and distributed in the reception room before they are discussed. The manuscript, for printing purposes, should not exceed 2,000 words, and must be accompanied by a short abstract both for the convenience of the Press at the Congress and for insertion, subject to the approval of the Council, in the Journal of the Institute, should it not be deemed desirable to publish the paper *in extenso*. No previously published paper can be accepted. The Council reserve the right of refusing any papers sent in ; and, in the case of those accepted, the discussion of them must depend on the time at the disposal of the meeting. Papers accepted for the Congress cannot be published by the authors, except by permission of the Council. The Council reserve to themselves the privilege of printing any paper, either wholly or in part, or of refraining from the publication thereof, if they see fit. MSS. should be forwarded by post as early as possible, and in any case not later than June 19, to the Secretary, The Royal Sanitary Institute, 90 Buckingham Palace Road, London, S.W.1.

### THE COLLEGE OF NURSING.

As a result of the collection made by the College of Nursing, the trained nurses and probationers of Great Britain and Ireland have presented to Princess Mary a writing set of four pieces in tortoise-shell and gold. The gift was handed to the Princess by Miss Tisdale, R.R.C., matron of the Hospital for Sick Children, Great Ormond Street, where Her Royal Highness worked as a nurse for some time.

At the Norfolk and Norwich Hospital, by kind permission, a lecture on " Some of Dickens' People " will be given on March 14 at 8 p.m., by Mr. M. M. Pattison Muir. Members of the College of Nursing free ; non-members' tickets, 1s.

